# Environmental Display Can Buffer the Effect of Pesticides on Solitary Bees

**DOI:** 10.3390/insects11070417

**Published:** 2020-07-05

**Authors:** Samuel Boff, Josué Raizer, Daniela Lupi

**Affiliations:** 1 University of Milan, Department of Food Environmental and Nutritional Sciences, 20133 Milan, Italy; daniela.lupi@unimi.it; 2 Universidade Federal da Grande Dourados, Faculdade de Ciências Biológicas e Ambientais, Dourados 79840-970, Brazil; jraizer@gmail.com

**Keywords:** artificial flowers, environmental change, foraging behaviour, fungicide, nectar uptake, network metrics

## Abstract

Environmental quality (e.g., diversity of resource availability, nesting sites, environmental display) plays an important role in an animal’s life. While homogeneous environments can restrict organisms from developing activities such as food seeking (behavioral impairment), more complex environments allow animals to perform activities with learning and behavioral perfecting outcomes. Pesticides are known to affect the learning and foraging behaviors of bees; however, little is known about the counterbalance displayed by the environment. Herein, we conducted two experiments that simulated distinct environmental displays, in which the effects of a fungicide (Indar^TM^ 5EW-febunconazole) on solitary bee foraging activities were tested. We found that the fungicide only impaired the activities of bees in one of the studied environments. The difference in visitation rates and flower exploitation of bees between the two different environmental displays led to changes in metrics of bee–flower networks across environments. Linkage density, a metric associated with pollination efficiency that is known to be impacted by different environments, differed across environments. Our results showed that ecological interaction network metrics can differ regarding the different environmental displays. This study indicates that environmental complexity helps balance the negative effects of pesticides on solitary bees and highlights the potential use of solitary bees as model organisms for experimental simulations of environmental change.

## 1. Introduction

Recent evidence has shown that environmental stressors such as deforestation, pathogens, exotic species, pesticides, lack of local resources and climate change negatively affect biodiversity [1,2] and can lead to changes in ecosystem functioning [3]. Along with common ecological indices used to measure diversity, quantitative measurements of behavior have also been proposed to estimate impacts of the environment/stressors on species [4]. Indeed, so called ethodiversity seems to be an important issue in ecology since behavioural changes may be a direct response to stress [5]. The presence/absence of one species in an environment, as well as environmental differences in local features, can alter many ecological dynamics, including network interactions [6,7]. Thus, the effect of stress on individuals and their behaviour might disrupt the stability of ecological dynamics, which is interesting for nature conservation [8].

Regarding environmental stressors, studies report that animals under stress can respond better in complex environments. It has been shown that individual foraging behaviour of fishes and crustaceans can be impaired by elevated CO_2_ levels, but may be buffered in complex environments when species of the same trophic level are added [9]. For mammals, environmental heterogeneity has been found to play a significant role in foraging activities of caged coyotes compared to free range animals [10]. Chickens placed in more heterogeneous environments with a feeder, wood shavings, and wooden block perches presented a more refined repertoire of behaviours than chickens placed in an area with a feeder and wood shavings only [11]. Zebra fishes needed additional time to perform activities and were more stressed in simple environments compared to individuals placed in enriched water tanks with the presence of plastic plants, plastic shelter, gravel substrate and a novel object rotated every week [12]. Differences in local environmental features have also been perceived in the foraging behaviour of free-range bees with different life history traits, which helps explain differences in bee–plant networks encompassing solitary and a social bee species [13]. 

While single stress sources can interfere with animal life (e.g., life span, foraging activities), the combined effects of multiple stressors can maximize negative effects, leading to the inability to be stimulated to perform sophisticated tasks such as foraging and food uptake. On a broader scale, such behavioral change due to stressors may affect the ecological interaction between distinct trophic-levels, such as those in bee–plant networks. Yet the variation in landscape features has been shown to affect network structures (e.g., linkage density), as well as pollination [7]. Pollination systems investigated in scenarios with bees exposed to pesticides resulted in reduced floral visitation rates and consequent reductions in fruit/seed production [14,15]. These findings may be explained by low flower exploitation [14,15] and poor pollination efficiency due to reduced ability to perform pollination [16]. Although studies reporting sub-lethal effects of pesticides have mostly focused on social bees, aspects of social interaction [17], foraging and memory ([18], but see [19]), and pollination itself [14,16], there is emerging evidence of behavioral impairment caused by pesticide exposure for solitary bees [20,21]. 

Considering that stressors may not impair animals in more complex environments and that animal behaviour in ecotoxicological and environmental contexts have rarely been analyzed together [8], we aimed to understand how bees exposed to a pesticide perform foraging behaviors in two different scenarios. Thus, we performed two experiments to test the effect of a low toxicity fungicide [22] on the foraging activities of a wild solitary bee in two different environments.

Herein, we hypothesized that the fungicide would affect (1) visitation rate and (2) nectar uptake performed by solitary bees. Additionally, we tested the hypotheses that (3) flower exploitation (number of flowers visited) decreases in bees exposed to pesticides leading them to have (4) different responses in different environment types for bee–plant networks. Since a more complex environmental display has been found to buffer the effect of stressors on foraging behavior [10,12], we predicted that different environment types affect solitary bee stimulus, leading to changes in floral visitation rates and bee–plant networks. Previous experiments with solitary bees have demonstrated their response to the presence of flowers in cage environments [23].

## 2. Material and Methods

### 2.1. Bee Species

*Osmia bicornis* is an univoltine solitary bee with wide distribution and is commonly found in Europe [24] where it is active at the beginning of the spring season and visits flowers during the summer. This species is an important pollinator of crops such as apples, cherries, plums [25] and is a generalist among solitary bees [24,26,27]. *Osmia bicornis* can be found in urban and non-urban environments and are attracted to human-made cavities [28]. In our experiments, we only used females from a single population (see Supplementary Material, Experimental details, for more information regarding the bee species) for two consecutive years.

### 2.2. Stressor

Theoretically, fungicides should not target insects, however, direct exposure [29,30] and indirect contact [31] have been shown to affect bees. Herein, we studied the effect of Indar^TM^ 5EW, Dow AgroSciences Italia S.R.L. Milano (Italy), a broad-spectrum fungicide with fenbuconazole 50 g/L as active compound, on the foraging behaviour of *O. bicornis*. This fungicide controls leaf spots, apple scab, pear scab and apple powdery mildew on apples and pears and is applied during flowering and fruiting seasons (Indar^TM^ 5EW, label). In 2010, the European Food Safety Authority (EFSA) reported that fenbuconazole has no effects on the mortality of honeybees [32] and an additional study conducted with honeybees and *Osmia cornifrons* highlighted its low toxicity in mortality tests [22]. In the current study, we worked with the field realistic dosage of Indar^TM^ 5EW. We reached field realistic dosage (product label) by diluting 1.4 L of Indar^TM^ 5EW (50 g of fenbuconazole per liter) in 1000 L of water (0.07 g de fenbuconazole per liter), which is 70 ng of fenbuconazole per microliter. See Supplementary Material, Experimental details, for more information regarding the stressor.

### 2.3. Training Phase

Bees learned that artificial flowers offered nutritional rewards and how to extract sucrose solution from them. During the training, we inserted newly emerged female bees (n = 45) into training cages (17 × 11.5 × 10 cm) individually. One artificial flower was placed at the centre of every cage. The flower consisted of a 1 cm^2^ coloured foam square with a 3 cm^2^ green piece of cardboard underneath to enhance floral contrast. In the middle, we placed a feeder that contained cotton imbibed with 25% sucrose solution from which bees could feed ad libitum. Bees remained inside the cage for two days and were observed feeding on this artificial flower.

### 2.4. Experimental Displays 

#### 2.4.1. Environment Type 1-Experiment with Flowers of a Single Colour Hanging on the Sides of the Arena

We simulated a lower diversity environment with eight artificial yellow flowers (see Training phase), filled with 20 µl of 25% sucrose solution that were hung on the sides of the flight arena (see Supplementary Material; Figure S1). After two days of training the bees (n = 18), they were starved for 1 h and randomly assigned to treatment (n = 9) and control (n = 9) groups on the third day. After placing a given bee in a queen marker cage, the bees in the control group received 1 µl of water on the thorax, while the treated bees received 1 µl of the field realistic dose (70 ng bee^−1^) of fenbuconazole on the thorax. Since these bees can forage in several hundred meters every day [33], which enhances their chances of coming in contact with agrochemicals on a daily basis, we exposed the bees to a daily treatment (chronicle exposure) by applying either water or fungicide to their thorax. After the exposure, each bee (control and treated bees) was individually released and observed three times (rounds) for 10 min inside a flight arena with an interval of 1 h between observations. Bees were observed for one to 4 days (see Supplementary Material; Table S1). After daily observations of foraging parameters, the bees were returned to the same training box with ad libitum sucrose solution available in the colored feeder. During every observation period, the following parameters were evaluated: the number of visits per bee (visitation rate), the number of flowers visited (flower exploitation) and the average time every bee spent for nectar uptake. Flower identity was recorded in connection with visits and this information was used to reconstruct networks (see below). A new set of artificial flowers were inserted into the cage before every new observation period to remove potential effect of previous visits on bee activity. A total of 1310 min of observation was carried out in the environment type 1 and this experiment was conducted between July-August 2018.

#### 2.4.2. Environment Type 2- Experiment with Flowers of Different Colours Set at the Bottom of the Arena

Since floral display [34] and different colour patterns can enhance floral visitation [35], and bees integrate stimuli from additional sensory modalities [36], we changed the display of the environment by placing 12 artificial flowers with two different colour types (6 yellow, 6 blue) at the bottom of the flight arena. Green cardboard was positioned below the artificial flowers to enhance contrast, and a semi-circular green cardboard was added inside the flight arena (see Supplementary Material; Figure S1). In the experiment with flowers of different colour types set at the bottom of the arena, control bees (n = 13) were trained either with blue (n = 7) or yellow flowers (n = 6) and treated bees (n = 14) were trained with blue (n = 8) or yellow flowers (n = 6). Bees were exposed to water or fungicide identically to the treatments in the environment type 1. Every single flower standing in the flight arena received 20 µl of sucrose solution at 25%. In addition to the number of flower visits per bee (visitation rate), flower exploitation and duration of nectar uptake, we also recorded information regarding the colour of the flowers visited (blue vs. yellow). Flower identity was recorded in connection with visits and this information was used to reconstruct bee–plant networks (see below). Bees were observed in two periods (rounds) of 20 min a day for 4 days, resulting in 40 min of observation per bee per day and a total of 3448 min. The experiment took place between April and May 2019.

The experiments were performed in two different years. It has been shown that changes in wintering duration affect body fat depletion and may be reflected in survival, however, this finding has been reported for a different species in North America exposed to different pre-wintering conditions [37,38,39]. Here, the pre-wintering conditions bees were exposed to were similar in these two years. Despite indications of changes in carbohydrate and lipid metabolism of extended post diapause wintering conditions, see [40], a negative effect of extended post diapause wintering conditions, especially on foraging behavior of *O. bicornis*, was not found. Moreover, the presence of *O. bicornis* constructing nests—where pollen from several plant species from hundreds of meters away was found during the summer [41]—seemed to mitigate possible drastic impairment of foraging for bees in this season.

### 2.5. Analysis of Foraging Behaviour

We treated our data based on direct observations and video recordings of the flight arena and the bees. In order to test the effects on number of visits and flower exploitation, we performed analyses of variance (ANOVA) and Tukey significant differences. Rounds in which individuals did not perform any visit (n_Environment type 1_ = 91, n_Environment type 2_ = 74) were excluded from the analysis. In ANOVA models, the independent variables were treatment (treated or control bees), day of observation, round of observation, and bee identity. Since environment type 2 included more flowers, we used the proportions of flowers in each round to consider the different number of flowers in the two environments regarding floral visitation rate and flower exploitation. This approach was taken as a proxy to visualize the effect of environment on bees in two independent experiments. Thus, the proportion of flowers used in the model for visitation rate was total number of flowers visited divided by the total number of flowers in the flight cage. Similarly, the proportion of flowers used for flower exploitation was the number of different flowers visited divided by the total number of flowers in the cage. All analyses were conducted in R language [42] and the partial residuals were verified with the car package in R [43].

### 2.6. Analysis of Nectar Uptake 

Nectar uptake, meaning the length of feeding time, was defined as the average time in which a bee drank nectar from flowers during the experiment. In order to test the effect of treatment on nectar uptake in environment type 1 and environment type 2, we used a Generalized Linear Mixed-Effects Model, GLMM [44] with feeding time as response variable and treatment and number of flower visits, as well as their interaction, were included as fixed factors. Bee identity was included as random factor in a model with normal distribution and a link log function.

### 2.7. Effect of Training Flower on Environment Type 2

The effect of flower training (blue or yellow) on bee flower colour nectar uptake preferences was tested in a model. To evaluate these preferences, the flower colour was included as a fixed factor and the ratio between the number of blue flowers and the total number of flowers as the response variable. The ratio varied between 0 and 1 (0 ≤ ratio _blue/(blue + yellow)_ ≤ 1); with values smaller than 0.5 indicating higher visitation in yellow flowers, values equal to 0.5 indicating nectar uptake was performed equally in blue and yellow flowers, and values higher than 0.5 indicating nectar was mostly collected in blue flowers. The bee identity, rounds, and day of observation were used as random factors with binomial distribution. Generalized linear mixed model analyses were performed in R using the lme4 package [44]. 

### 2.8. Network Analysis

Quantitative matrices of interactions were built independently for the environment type 1 and environment type 2 for different rounds of observations on a given day of experiments with bee identity in rows and flower identity organized in columns. This generated multiple networks from both environment types (n_Environment type 1_ = 10 networks, n_Environment type 2_ = 8 networks). The network interaction analyses were built using the igraph package [45] in R, and interaction network metrics were acquired using the bipartite package [46]. First, we used individual level specialization metrics (d’) to detect individual bees specialized on individual flowers. To compare network-level bee–flower structure metrics of 18 networks from two environment types, we analyzed connectance, nestedness (NODF), linkage density, and complementary specialization (*H*_2_*’*) due to robustness of these metrics in ecological networks to environmental changes and perturbations [47]. The connectance represented the number of observed interactions from all possible network interactions [48]. Nestedness, measured with NODF, resembles a metric based on overlap and decreasing fill [49]. The linkage density was calculated to provide means of changes in the structure of the network since it measures the average number of interactions per specimen in the web [50]. Complementary specialization (*H*_2_*’*), was used to measure the degree of specialization in the network [51]. 

Firstly, for species-level specialization index d′ with assignment of treatment, we measured the dependence of bees on a specific flower(s). In this model (GLMM), a measurement of specialization index (d’) was the response variable; treatment, training flower, and environmental type were used as fixed factors. Bee identity, round and day were included as random factors with a binomial model distribution. Secondly, from the network-level structural metrics obtained per experiments and days, we performed Linear Mixed Models with connectance, NODF, linkage density and *H*_2_*’* as response variables; environment type as a fixed factor; and round and day as random factors. A normal distribution was used in all models, except for *H*_2_*’,* which used a binomial distribution. Both indexes of specialization, d’ and complementary specialization *H*_2_*’* range from 0 to 1, with 0 indicating completely random interaction partners and 1 indicating complete specialization [52]. 

We used the ‘DHARMa’ package with a simulation-based approach to access interpretable scaled (quantile) residuals for fitted (generalized) linear mixed models [53]. Only models with non-significant deviation from observed and expected residuals were used in the analysis.

## 3. Results

Bees exposed to fenbuconazole displayed a different foraging behaviour compared to control bees in environment type 1. However, control and treated bees in environment type 2 performed foraging similarly between them (see Supplementary Material; Figure S2A,B).

### 3.1. Environment Type 1

**Figure 1:**
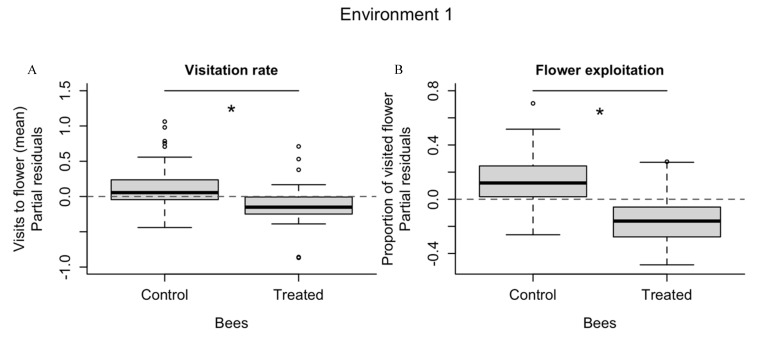

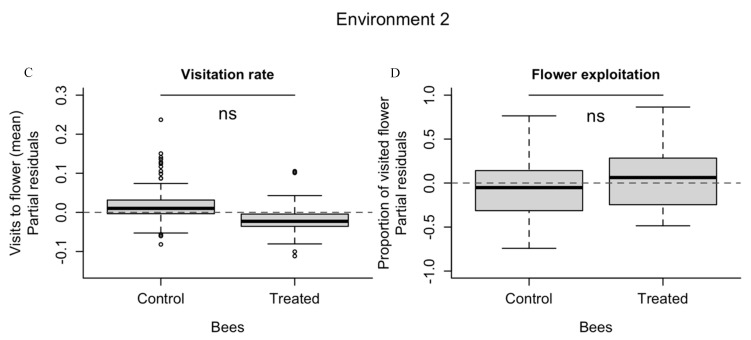
The solitary bee *Osmia bicornis* responded negatively to pesticide exposition in environment type 1. The visitation rate and the flower exploitation (**A**,**B**) of this bee species was significantly reduced after exposure to fenbuconazole compared to the control group. The negative effect was not observed in environment type 2 (**C**,**D**). * Statistically different (α = 0.05); ns = not significant.

Bees exposed to fungicide visited significantly less flowers (n_Flowers visited_ = 70) than control bees (n_Flowers visited_ = 155) (F_1, 108_ = 4.55, *p* = 0.035, Figure 1A, Supplementary Material; Figure S2A) throughout the experiment. There was an increase in the mean number of visitations throughout the study, which was significantly lower on the first day (F_3, 108_ = 7.86, *p* < 0.001, and Tukey HSD = 0.28, *p* < 0.025) and greater for control bees than for treated ones during the first round (see Supplementary Material; Figure S2A). Regardless of treatment, the number of visits did not differ throughout the daily rounds (F_2, 108_ = 1.75, *p* = 0.178) but differed among bees (F_16, 108_ = 7.11, *p* < 0.001). See Supplementary Material, Figure S3.

Flower exploitation in the environment type 1 differed significantly between treatment and control groups. Treated bees visited a lower proportion (n = 29) of flowers than control bees (n = 83) visited (F_1, 108_ = 10.76, *p* = 0.001, Figure 1B, Supplementary Material; Figure S2B). Although there was a daily increase in the proportion of flowers visited, this difference was significantly smaller on the first day only (F_3, 108_ = 9.06, *p* < 0.001, and Tukey HSD < 0.24, *p* < 0.002). The proportion of flowers visited did not differ during the three rounds of the experiment (F_2, 108_ = 1.253, *p* = 0.289), but did differ among bees (F_16, 108_ = 6.81, *p* < 0.001). See Supplementary Material;Figure S4.

### 3.2. Environment Type 2

Regarding environment type 2, the number of visits throughout the entire experiment between treated (n = 676) and control group (n = 920) did not differ significantly (F_1,143_ = 1.742, *p* = 0.188), although the difference observed between the two groups presents an interesting trend (Figure 1C, Supplementary Material; Figure S2A). Daily exposition and different rounds did not play a significant role in the number of visits (F_3, 143_ = 1.965, *p* = 0.121 and F_1, 143_ = 2.020, *p* = 0.157, respectively). At first glance, the bees trained on blue flowers (regardless of treatment) presented better visitation rates, but the difference was not statistically supported (F_1, 143_ = 0.012, *p* = 0.911). In fact, only bee identity explained variation in visits. See Supplementary Material, Figure S5.

Flower exploitation by *O. bicornis* in the environment type 2 did not differ between fungicide treatment (n = 250) and control (n = 265) (F_1, 143_ = 0.04, *p* = 0.85, Figure 1D, Supplementary Material; Figure S2B), throughout the days and rounds (F_3, 143_ = 1.06, *p* = 0.37; F_1, 143_ = 0.460, *p* = 0.50). The colour of the training flower, blue vs. yellow (F_1, 143_ = 2.83, *p* = 0.09), and bee identity (F_24, 143_ = 0.69, *p* = 0.85) did not have a significant effect (see Supplementary Material, Figure S6). The proportion of blue flowers visited by *O. bicornis* did not differ between control and treated bees (F = 0.037 *p* = 0.85) throughout the days and rounds (F_3, 143_ = 2.50, *p* = 0.06, F_1, 143_ = 1.941, *p* = 0.17). Although bees that trained with blue flowers visited a higher number of blue flowers (n = 719) than bees trained with yellow flowers (n = 332), the proportion of blue flowers visited by bees did not differ significantly regarding the colour bees were trained with (F_1, 143_ = 2.61, *p* = 0.11). Again, only bee identity explained the proportion of blue flowers (F_24, 143_ = 2.64, *p* < 0.001). See Supplementary Material, Figure S7.

### 3.3. Nectar Uptake

There was no significant effect of interaction between the number of visits and treatment on length of feeding time in environment type 1 (GLMM, χ^2^ = 2.85, df = 1, *p* = 0.09). In environment type 2, the effect of the same interaction on length of feeding time remained non-significant (GLMM, χ^2^ = 0.21, df = 1, *p* = 0.64). Moreover, the training flower did not significantly affect the ratio of flower colours a given bee visited and performed feeding on (GLMM, nχ^2^ = 1.8343, df = 1, *p* = 0.175).

### 3.4. Individual Specialization and Interaction Network Structures

**Figure 2:**
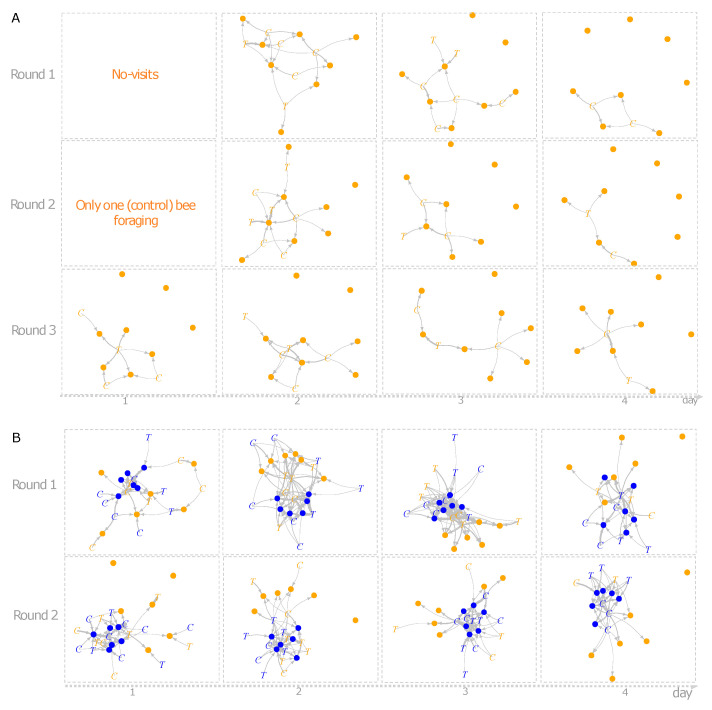
Network interactions of O. bicornis on artificial flowers in environment type 1 (**A**) and environment type 2 (**B**). Bees are indicated by “C” and “T” (control and treated with fenbuconazole, respectively) and the colour of the letter indicates the training phase (blue or yellow flower) of a given individual. Coloured dots indicate flowers in both experiments and thickness of links indicates the number of interactions.

**Figure 3:**
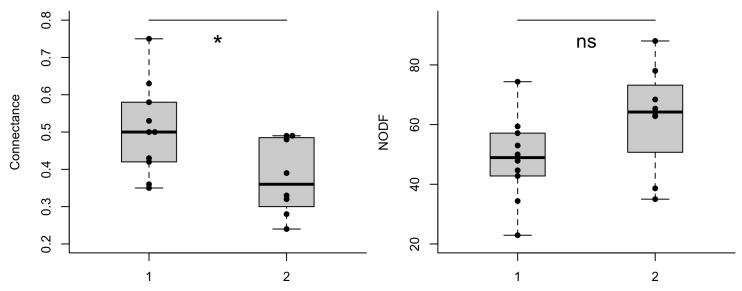

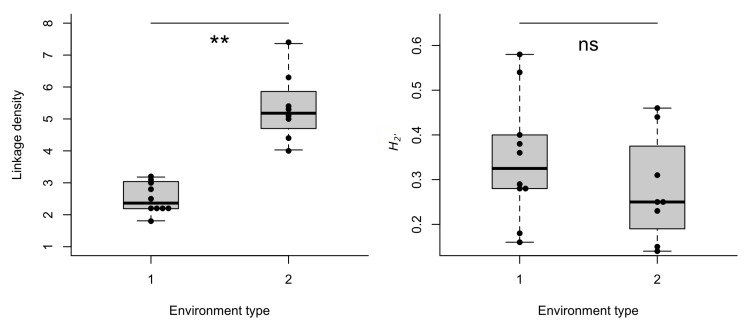
Ecological metrics of networks established from bees (*O. bicornis*) and flowers (artificial flowers). Measures were recorded in flight cages simulating two different environment types (1 and 2, see methods). * *p* < 0.01, ** *p* < 0.001, ns = non-significant.

The individual d’ specialization index did not differ between treatments, experiments, time of observation or training flower (GLMM’s χ^2^_treatment_ = 0.037, df = 1, *p* = 0.846; χ^2^_experiment_ = 0.059, df = 1, *p* = 0.813; χ^2^_time of observation_ = 0.418, df = 1, *p* = 0.517; χ^2^_training flower_ = 0.069, df = 1, *p* = 0.791). Yet, the network structures generated in both experiments differed notoriously (Figure 2A,B). The number of interactions (links) in the first experiment was on average smaller (11 ± 5.03 interactions) than the observed number of interactions in the second experiment (56 ± 18.6). Some of the metrics generated from group level analysis differed between environment type 1 and environment type 2. Connectance was significantly higher in environment type 1 (lmer, χ^2^ = 6.85, df = 1, *p* = 0.008); and the difference in NODF, a measure of nestedness, was borderline significant between the environments (lmer, χ^2^ = 3.40, df = 1, *p* = 0.06). Linkage density (lmer, χ^2^ = 73.63, df = 1, *p* < 0.001) differed significantly between environments, while the difference in complementary specialization *H*_2_*’* was not statistically different between environments type 1 and type 2 (lmer, χ^2^ = 1.15, df = 1, *p* = 0.28), see Figure 3.

## 4. Discussion

Pesticides and environmental homogenizations are among the major threats to bees [54]. Here, we showed that the solitary bee *O. bicornis* can respond differently to an agrochemical stressor depending on the arrangement of its environment. While there is evidence that a mixture of insecticide and fungicide leads to abnormal behaviors in bees, e.g., [30,55], the single effects of fungicide on bees has not been analyzed extensively. Herein, we accept the hypotheses that pesticides affect foraging behavior of bees dependent on environmental display and this change affected structural metrics of networks such as connectance and linkage display. Therefore, we reject the hypothesis of impact on nectar uptake, as well as the effect of the flower training and specialization. We found that a low toxicity fungicide (Indar^TM^ 5EW-febunconazole) reduced the foraging behavior of bees placed in an environment with eight flowers of a single colour hanging in the sides of the arena. This impact was not observed in an environment with 12 flowers of different colours set at the bottom of the arena. On a greater scale, the continued use of combined stressors (fungicide plus environmental display) could negatively impact ecosystem services by altering metrics of bee-plant interactions.

The environments studied here (environment type 1 and environment type 2) simulated two distinct environments with differences in displays that are more commonly observed in monoculture fields (i.e., a homogeneous environment) compared to non-anthropic environments (i.e., higher diversity of floral resources). Sattler et al. (2020) observed differences in species composition and functional diversity of pollinators when studying the combined effects of pesticides and environmental heterogeneity in rice fields [56]. Along with species composition, our results, which focus on behavioral changes that depend on environmental features, seem to empirically match the “non-regret measures” for safeguarding pollinators presented in the “roadmap for insect conservation and recovery”, when it comes to the impact of pesticides and environmental change on pollinators [54].

### 4.1. Environment Type Effect on Foraging Behaviour of Bees

Herein, we show that the low toxicity pesticide can affect solitary bee foraging behavior by reducing flower visitation. However, the negative effect that was observed directly after application of the agrochemical decreased over time, with similar visitation rates between control and treatment groups after 1 and 2 h of fungicide (2nd and 3rd rounds) application. The disappearance of pesticide toxicity on bees has also been observed in a social interaction context [17].

Interestingly, no differences were observed between treated and control group in environment type 2. Increasing the number of flowers and adding a mix of flower types buffered the negative impact on foraging behaviour that was observed in the environment type 1. The flower visitation rate of treated bees foraging in the environment type 2 did not differ from the control group, which was much higher for both compared to bees in the environment type 1.

Environmental heterogeneity has been shown to underpin changes in behaviour due to plastic response to an environment. For example, the ability to perform a new repertoire of behaviours can occur due to changes in neuronal functioning driven by environmental stimulation [57]. As a consequence of neuronal changes, biotic and abiotic stimulus can play an important role in animal physiology [57], which has been linked to nerve excitement, change in respiratory patterns, and water loss in different bee species [58,59,60]. Additionally, enhancement in the number of flowers and flower type can play a role in sensory cues [36,61], which seemed to optimize resource exploitation by treated bees herein.

In open sites, ability to find flowers depends on the mobility of pollinators, which has been hypothesized to determine the ratio of pollination success [62] and, therefore, increase plant genetic diversity [63]. This occurs because several wild and crop plant species rely on cross pollination, i.e., depend on pollen grains that originate from other, non-parental individuals for seed and fruit production. According to our results, environment enrichment by increasing aspects of floral diversity resulted in higher visitation rates. In nature, environments presenting diversified floral characteristics have been shown to benefit the reproductive output of plants and can result in greater yields [64]. Ultimately, it can also ameliorate the reproductive output of pollinators by increasing offspring survival and nest development. Recent studies focusing on the development of bee broods in natural conditions, e.g., [65,66], have shown that a wider diet breadth, which likely relies on flower exploitation, enhances the possibility of a balanced diet; an important role in brood nutrition which can trigger physiological and immunological changes in bees [67].

### 4.2. Nectar Uptake and Effect of Training Flower

Agrochemicals such as neonicotinoid pesticides are known to impair feeding in social bees [68,69], as well as in solitary bees when pesticide exposure occurs with a fungicide [30]. However, these impairments were recorded when agrochemical exposure was administered orally. Herein, despite its chronical administration, the fungicide was exposed to the thorax. Thus, the fungicide used here, which is known for its low toxicity, does not seem to strongly affect feeding.

For social bees, it is known that pre-training on coloured flowers can set their foraging preferences [70]. Here, the effect of flower training did not significantly explain flower preferences of bees performing nectar uptake during foraging events. Both bees trained with blue and yellow flowers foraged mostly on blue flowers (average ratio > 0.5, see methods), but differences were not significant. Although memory can be harmed with high toxicity pesticide [68], topical exposure of fenbuconazole did not seem to affect memory of *O. bicornis*. Therefore, the effect of fenbuconazole on nectar uptake and memory remain unknown when it comes to oral exposure.

### 4.3. Individual Specialization and Structural Network Metrics

The different proportions of visited flowers presents an opportunity to understand how combined factors (pesticide + environmental types) affect interaction network metrics. The observed changes on behaviors of the control and fungicide treatment groups of *O. bicornis* in the two different environmental experiments provided means to understand metrics associated with specialization and those associated with structure of ecological network. Specialization did not differ at individual (d’) or at network-level (*H*_2_*’*). Similar d’ values indicate that flower visitation attempts in both environments occurred randomly, reinforcing that associations due to experience and learning were not harmed, which is similar to results found for social bumble bees [19].

The variation observed in the foraging behaviour of bees lead to more evident changes in structural network metrics. Simulating different environments supported field level studies, showing that the structure of bee–plant networks depends on local environmental features [7,13,71]. Herein, the overall number of interactions in environment type 1 was always lower than in environment type 2. Interestingly, adding flowers to the experimental setting enhanced bee–flower interactions. While the number of unvisited flowers was higher in environment type 1, it remained low in environment type 2. Linkage density, the ratio between the number of individuals and the number of links observed between individual bees, provided the mean links per bee in the networks. At field level, enhancing the number of flowers has been shown to increase visitation rate and success in pollination by solitary bees probably due to flower exploitation, a prerequisite for reproduction in cross pollinated plant species [62]. In a network context, a positive association between increased linkage density and pollination was also found [7,72]. Thus, if our findings are applied to pollination systems in more diverse agricultural environments, adding flower types (e.g., wildflowers strips) rather than maintaining monocrops, would likely provide a higher yield if flower visitation rate is performed by an efficient pollinator [64,73].

NODF, a metric of nestedness, was borderline significant between environments. Thus, the higher NODF in environment type 2 is noteworthy. In networks with multiple species, more than 60% of nestedness in the networks can be explained by relative abundance of species [74]. In our study, increasing the number of flower types may have led to higher NODF in the environment type 2 [47]. The borderline significance may exemplify the need to investigate such effects on new experiments by comparing environments with more diversified flower types.

In our study, increasing the number of flowers in environment type 2 allowed bees to have new interactions. Despite the enhancement of new interactions (number of links) compared to environment type 1, the total number of possible interactions in environment type 2 was not reached, culminating in a smaller connectance index in environment type 2 compared to environment type 1. Using a controlled number of flowers in both environments highlighted that a positive association of connectance and pollination success on natural environments may be misleading. The environment type 2 displayed many more interactions (average 56) than environment type 1 (average 11). This means that in open environments our results would likely reflect a higher index of plant fertilization in environments that are more similar to environment type 2, considering equal efficiency of the bee as pollinator when visiting flowers in both environments. A future study in which network size is kept constant (e.g., same number of bees and flowers) would help test differences in network structures and pollination across environments.

The stimuli-response of solitary bees to environmental complexity in field and lab-based experiments showed these pollinators are suitable for free range or cage studies; a fact that has often been reported for vertebrates [75]. This seems to indicate that solitary bees, e.g., *O. bicornis* could potentially be used as model organisms in experimental simulations of environmental change, in addition to more commonly used social bees (e.g., honey bees and bumble bees). Our findings showed a higher impact of the pesticide in one environment rather than both tested, which is as an important topic to consider in conservation plans for solitary bee species, especially because this bee group is so important for pollination. Moreover, further studies determining how pesticide-environment affects both solitary and social bees are needed. Herein, from a conservation perspective, the foraging differences between treated and control bees provided a meaningful use of ethodiversity with solitary bees when comparing behavioral changes of bees exposed to multiple stressors [4,8].

## 5. Conclusions

Our findings showed the impairment of bee foraging behaviors due to pesticides, corroborating results from field level studies. Therefore, our results suggested that the effect of low toxicity pesticides in the field depend on environmental display including flower availability. Regarding integrated pest management, a higher diversity of flowers might be advantageous for bees, as well as crop production, since it can enhance visitation rates and also decrease risks of energetic stress for bees. Future work should test if differences in the environment buffer behavioral impairment caused by pesticide application in solitary bees at semi-field levels.

## Supplementary Materials

The following are available online at https://www.mdpi.com/2075-4450/11/7/417/s1, Figure S1: Experimental flight cages, Figure S2: Visualization of visitation rate and exploitation per individuals, Figure S3: Complete panel with results of all models, Table S1: Experimental details.
