# Supplementary material for: Environmental Display Can Buffer the Effect of Pesticides on Solitary Bees
Source: Insects. 2020 Jul 5;11(7):417. doi: 10.3390/insects11070417 (PMC7412123; doi:10.3390/insects11070417)
Supplement: Supplementary file 1 [file insects-11-00417-s001.pdf]

Supplementary material

Experimental details

Osmia bicornis

Osmia bicornis cocoons were obtained in 2017 and 2018 from a regularly maintained stock population located in the Botanical Garden of the Martin Luther University Halle-Wittenberg (Halle/Saale, Germany). The cocoons were stored in the fridge (4 °C) until needed. Bees were sexed according to the hair colour of the clypeus by gently opening the cocoons at the top and only the females were used in the experiment. The bees used in the experiment were fixed at the end of the trials.

Stressor details

Fenbuconazole is used mainly as part of a routine preventative spray program. This fungicide was developed for controlling leaf spot, apple scab, pear scab and apple powdery mildew on apples and pears and is applied during flowering and fruit season (Indar™ 5EW, label). This compound is a racemic mixture of two enantiomers that belongs to the triazole fungicides family. It targets an enzyme (C14-demethylase) involved in the biosynthesis of ergosterols, causing a toxic accumulation of methylated sterols in fungi tissues. According to EFSA (European Food Safety Authority), this compound persists in the environment for a long-period and can contaminate aquatic environments through run-off.

Table S1. Number of individuals used in the experiment with environment type 1 and type 2

Environment type 1	Day 1	Day 2	Day 3	Day 4
Round 1	18	15	11	4
Round 2	16	14	10	4
Round 3	16	12	9	4
Environment type 2	Day 1	Day 2	Day 3	Day 4
Round 1	26	24	21	15
Round 2	25	24	20	14
